# The complete mitogenome of *Habropoda rodoszkowskii* (Hymenoptera: Apidae) and phylogenetic analysis

**DOI:** 10.1080/23802359.2020.1773954

**Published:** 2020-06-04

**Authors:** Huanhuan Lu, Dunyuan Huang

**Affiliations:** Chongqing Key Laboratory of Vector Insects, College of Life Sciences, Chongqing Normal University, Chongqing, China

**Keywords:** *Habropoda rodoszkowskii*, Hymenoptera, mitogenome, phylogeny

## Abstract

The mitogenome of *Habropoda rodoszkowskii*, the first complete mitogenome sequence of the genus Habropoda (hymenoptera: Apidae), was sequenced. The mitogenome is 18,497 bp (The proportion of A + T in 80.7%) long, with 37 classic eukaryotic mitochondrial genes (including 13 PCGs, 22 tRNAs, and 2 rRNAs) and an AT-rich region (The proportion of A + T in 78.2%). The Bayesian-inference and Maximum-likelihood phylogenetic relationship was constructed using 15 species from Hymenoptera. According to the phylogenetic tree, *Habropoda rodoszkowskii* converges with genus *Nomada* bees (*Nomada flava and Nomada flavoguttata*) to be supported. In addition, *Habropoda rodoszkowskii* is more closely related to Apidae than to Megahilidae and Colletidae.

*Habropoda rodoszkowskii* belongs to the genus *Habropoda* within the family Apidae. This species is distributed domestically in Zhejiang, Yunnan, Tibet, and abroad in Sikkim, Nepal, India. In 1896, the species was named by Dalla Torre (1896). In 2000, Chinese scientist Wu Yanru made a detailed morphological description of the species (Wu 2000). To date, our research team has sequenced the complete mitogenome of *H. rodoszkowskii* for the first time, which was collected for the first time in Jinshi Town, Xiangtan City, Hunan Province, China (N 27.990146, E 112.409546). The samples used in the experiment are now stored in the Chongqing Key Laboratory of Vector Insects, Chongqing Normal University (Accession number: LHH-2018-HBHTF-1) and the GenBank accession No. is MT436266.

The complete sequence of *H. rodoszkowskii* was 18,497 bp, the content of A + T was 80.7%, and it contained 13 protein-coding genes (PCGs), 22 tRNAs, 2 rRNAs, and an AT-rich region (Control region, CR). Compared to other Hymenoptera (Zhao et al. 2017; Zheng et al. 2018), the mitogenome of *H. rodoszkowskii* has similar gene content, order and orientation. In addition, 20 intergenic regions and 14 overlapping regions are scattered throughout the genome.

The starting codon of 13 PCGs is in the form of ATN (including six ATT, five ATA, and two ATG). They end in three termination codons (including 11 TAA, one TAG and one T––). 22 tRNA genes have a cloverleaf structure except for trnS2, whose dihydrouridine (DHU) arm forms a simple loop and the phenomenon has been reported in other Hymenopteran species (Wei et al. 2010; Huang et al. 2016). The 16S rRNA (rrnL) and 12S rRNA (rrnS) genes are located on the minority stand. At the same time, the two genes are separated by trnV, which is common in the mitogenomes of Hymenoptera (Shi et al. 2015; Wang et al. 2020). The AT-rich region (CR) was 2882 bp long.

To describe the position of *H. rodoszkowskii* in the hymenoptera phylogenetic network, 15 complete hymenoptera mitogenomes were downloaded from GenBank. Furthermore, *Abispa ephippium* and *Philanthus triangulum* were used as the outgroup. Phylogenetic trees were constructed from the 13 PCGs using two methods: Bayesian-inference (BI) phylogenies were inferred using MrBayes 3.2.6 (Ronquist et al. 2012) and maximum-likelihood (ML) phylogenies were inferred using IQ-TREE 1.6.8 (Nguyen et al. 2015).

Two phylogenetic trees have the identical topological structure and each node has high supports (Figure 1). The phylogenetic hierarchy of various organisms in the phylogenetic tree is consistent with previous studies (He et al. 2018). It can be seen from the phylogenetic tree that *H. rodoszkowskii* converges with genus *Nomada* bees (*Nomada flava and Nomada flavoguttata*) to be supported. In addition, *H. rodoszkowskii* is more closely related to Apidae than to Megahilidae and Colletidae. This mitogenome data can be further applied to DNA barcode technology and the establishment of phylogenetic relationships among other species.

**Figure 1.**
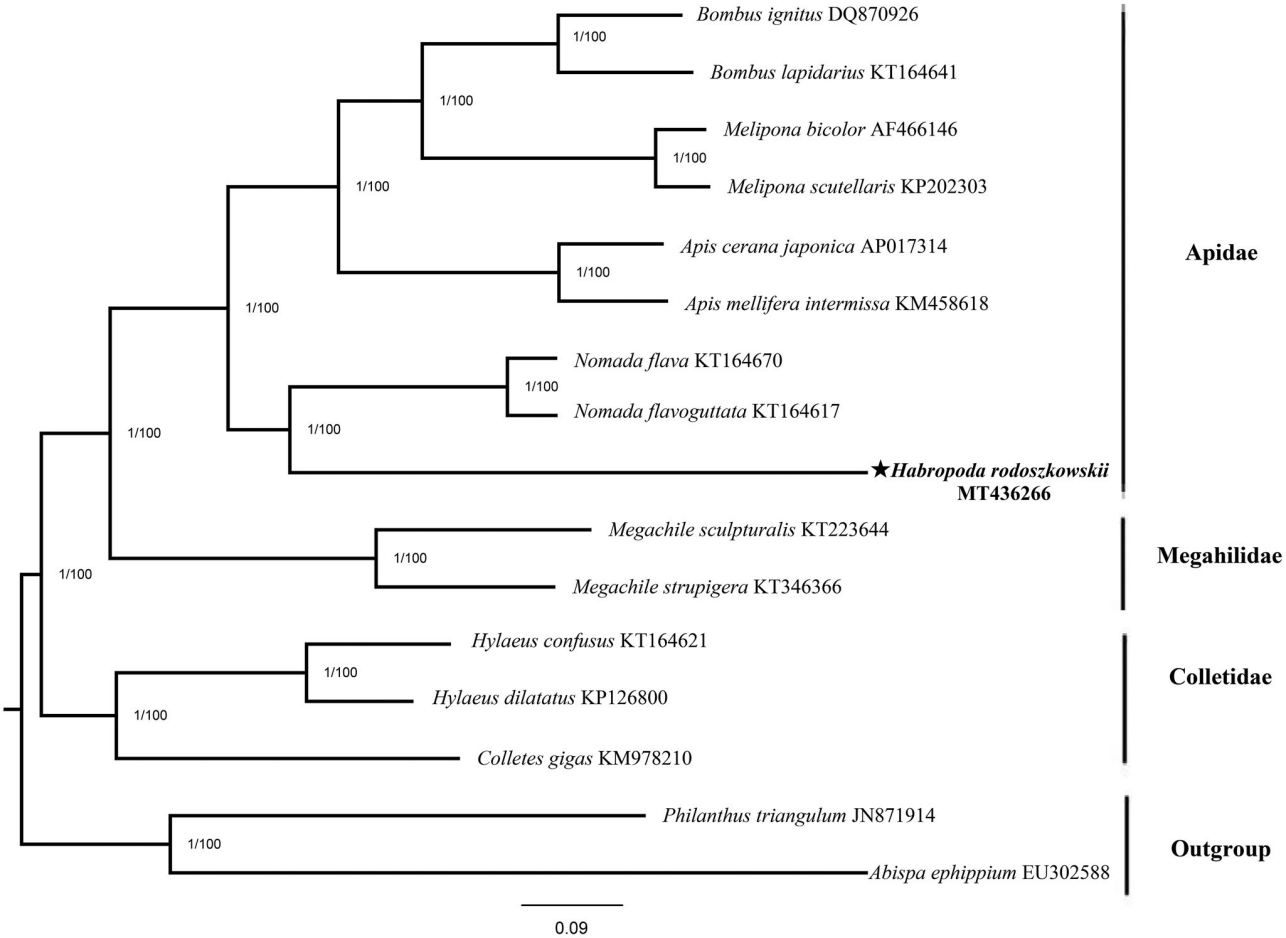
Phylogenetic tree of *H. rodoszkowskii* was constructed using 13 PCGs of mitogenome from Hymenoptera species and two outgroups. The support values of the corresponding nodes are shown above (left is bootstraps of BI, right is posterior probability of ML).

## Data Availability

The mitochondrial genome data of 16 species are used in the article. These data come from the following resources in the public domain: National Center for Biotechnology Information, https://www.ncbi.nlm.nih.gov/, and the GenBank No. of each species has been marked in Figure 1.

## References

[CIT0001] Dalla Torre K. V. 1896. *Podalirius radaskowskii* Dalla Torre. Cat Hym. 10:285.

[CIT0002] He B, Su TJ, Wu YP, Xu JS, Huang DY. 2018. Phylogenetic analysis of the mitochondrial genomes in bees (Hymenoptera: Apoidea: Anthophila)). PLoS One. 13(8):e0202187. 30092091 10.1371/journal.pone.0202187 PMC6084986

[CIT0003] Huang DY, Su TJ, He B, Gu P, Liang AP, Zhu CD. 2016. Sequencing and characterization of the *Megachile strupigera* (Hymenoptera: Megachilidae) mitogenome. Mitochondrial DNA Part B. 1(1):282–311. 33537412 10.1080/23802359.2016.1166078 PMC7831656

[CIT0004] Nguyen LT, Schmidt HA, von Haeseler A, Minh BQ. 2015. IQ-TREE: a fast and effective stochastic algorithm for estimating maximum-likelihood phylogenies. Mol Biol Evol. 32(1):268–274. 25371430 10.1093/molbev/msu300 PMC4271533

[CIT0005] Ronquist F, Teslenko M, van der Mark P, Ayres DL, Darling A, Höhna S, Larget B, Liu L, Suchard MA, Huelsenbeck JP. 2012. MrBayes 3.2: efficient Bayesian phylogenetic inference and model choice across a large model space. Syst Biol. 61(3):539–542. 22357727 10.1093/sysbio/sys029 PMC3329765

[CIT0006] Shi QH, Sun XY, Wang YL, Hao JS, Yang Q. 2015. Morphological characters are compatible with mitogenomic data in resolving the phylogeny of *Nymphalid butterflies* (Lepidoptera: Papilionoidea: Nymphalidae). PLoS One. 10(4):e0124349 25860387 10.1371/journal.pone.0124349 PMC4393276

[CIT0007] Wang CY, Zhao M, Xu HL, Zhang FL, Zhong YH, Feng Y, Wang SJ. 2020. Complete mitogenome of the stingless bee *Lepidotrigona terminata* (Hymenoptera: Meliponinae) and phylogenetic analysis. Mitochondrial DNA Part B. 5(1):752–753. 33366734 10.1080/23802359.2020.1715298 PMC7748534

[CIT0008] Wei SJ, Tang P, Zheng LH, Shi M, Chen XX. 2010. The complete mitochondrial genome of Evania appendigaster (Hymenoptera: Evaniidae) has low A + T content and a long intergenic spacer between atp8 and atp6 . Mol Biol Rep. 37(4):1931–1942. 19655273 10.1007/s11033-009-9640-1 PMC2831182

[CIT0009] Wu YR. 2000. Hymenoptera Melittidae Apidae. In: Fauna Sinica, Insecta. Vol. 20. Beijing: Sci. Press; p. 324–325. (in Chinese).

[CIT0010] Zhao X, Wu Z, Huang J, Liang C, An J, Sun C. 2017. Complete mitogenome of *bombus breviceps* (hymenoptera: apidae). Mitochondrial DNA Part B. 2(2):604–606. 33473917 10.1080/23802359.2017.1372710 PMC7800184

[CIT0011] Zheng BY, Cao LJ, Tang P, Achterberg KV, Hoffmann AA, Chen HY, Wei SJ, Chen XX. 2018. Gene arrangement and sequence of mitochondrial genomes yield insights into the phylogeny and evolution of bees and sphecid wasps (Hymenoptera: Apoidea)). Mol Phylogenet Evol. 124:1–9. 29510236 10.1016/j.ympev.2018.02.028

